# Transformative approaches in hemophilia management: from traditional therapies to prenatal stem cell treatment 

**DOI:** 10.3389/fbioe.2025.1684096

**Published:** 2025-11-21

**Authors:** Yung-Tsung Kao, Hueng-Chuen Fan, Gary Ro-Lin Chang, Jen-Kun Chen, Chih-Ching Yen, Muhammad Sufian, Shang-Hsun Yang, Chuan-Mu Chen

**Affiliations:** 1 Department of Life Sciences, and Doctorial Program in Translational Medicine, National Chung Hsing University, Taichung, Taiwan; 2 Ph.D. Program in Tissue Engineering and Regenerative Medicine, National Health Research Institutes and National Chung Hsing University, Taichung, Taiwan; 3 Department of Pediatrics, Tungs’ Taichung Metroharbor Hospital, Taichung, Taiwan; 4 Institute of Biomedical Engineering and Nanomedicine, National Health Research Institutes, Miaoli, Taiwan; 5 Department of Internal Medicine, China Medical University Hospital, and China Medical University, Taichung, Taiwan; 6 Institute of Molecular Biology and Biotechnology (IMBB), The University of Lahore, Lahore, Pakistan; 7 Department of Physiology, Institute of Basic Medical Sciences, National Cheng Kung University, Tainan, Taiwan; 8 The iEGG and Animal Biotechnology Center, Rong Hsing Research Center for Translational Medicine, National Chung Hsing University, Taichung, Taiwan; 9 Center for General Educational, National Quemoy University, Kinmen, Taiwan

**Keywords:** hemophilia, gene therapy, bypassing therapy, *in utero* stem cell therapy, prenatal treatment

## Abstract

The increased global incidence of hemophilia, along with its concomitant consequences arising from prolonged hemorrhage after an injury and a heightened vulnerability to internal bleeding in joints or the brain, brings to the forefront the development of innovative therapeutic strategies to alleviate this hereditary genetic disorder. Current treatments for hemophilia primarily depend on plasma concentrates; however, their widespread use is constrained by the reliance on blood donations and the associated risk of infections. Alternative protein-based therapeutics, such as recombinant coagulation factors, bypassing agents, and non-factor-based drugs, have been approved and utilized for the prophylaxis and treatment of hemophilia with promising outcomes; yet, their shortcomings consist of the necessity for repeated administration and the likelihood of inhibitor formation. With the emergence of the cutting-edge gene editing (CRISPR/Cas9) and gene delivery (viral and non-viral vectors) techniques, the recent progression in gene therapy, stem cell transplantation, and prenatal interventions via *in utero* stem cell therapy inspires optimism for individuals afflicted with hemophilia and their families, even though these innovative techniques remain in the preclinical stage with a lot of technical and ethical issues needing to be resolved. This review article provides a comprehensive overview of hemophilia management, from traditional therapies to advanced prenatal stem cell treatments, highlighting the evolution and future directions in addressing this genetic bleeding disorder.

## 1 Introduction

**FIGURE 1:**
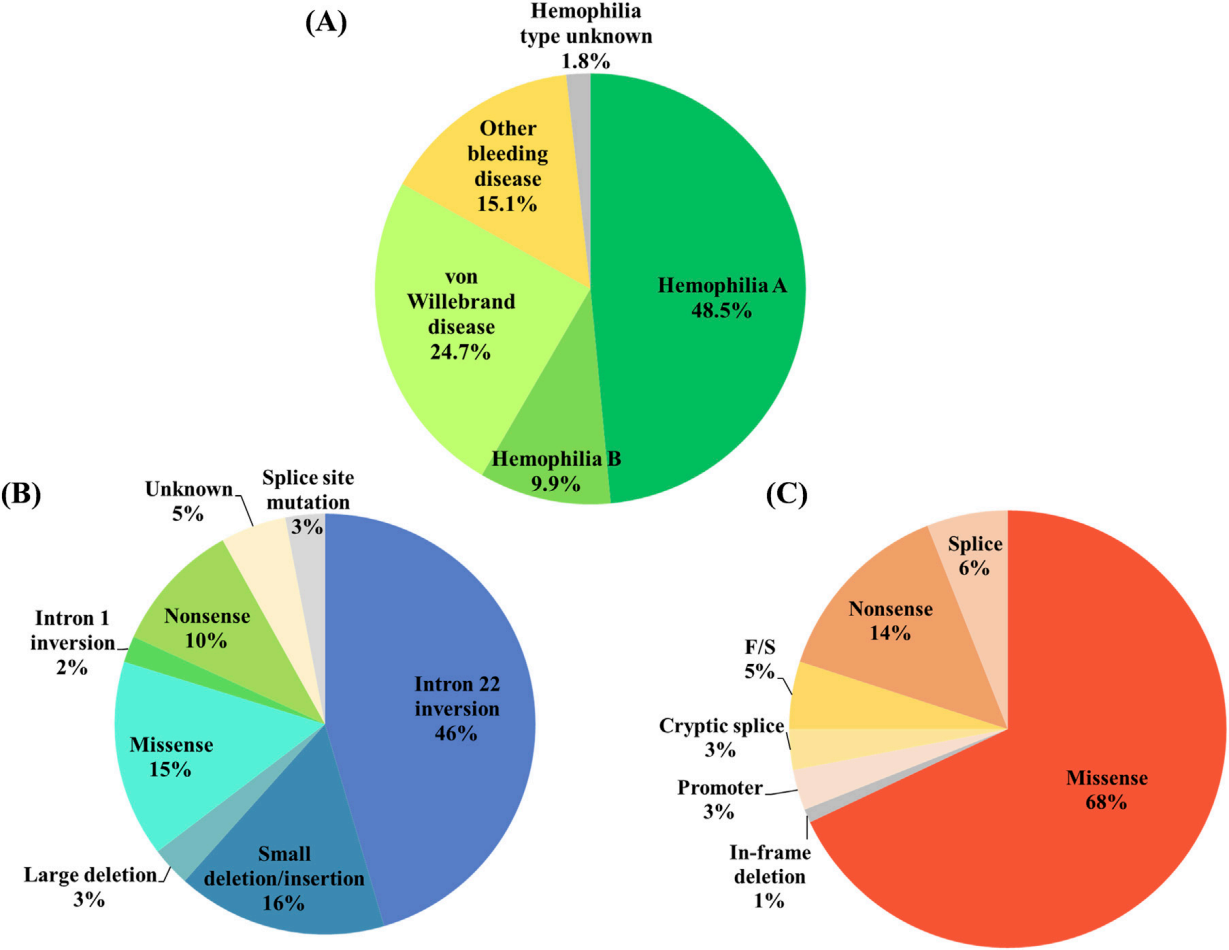
Proportional distributions pertaining to bleeding disorders and genetic variations associated with hemophilia. **(A)** Proportional distributions of prevalent bleeding disorders. Hemophilia A is deemed the most widespread coagulation disorder, noted for its nearly 50% prevalence among diverse bleeding disorders. **(B)** Hemophilia A‒associated genetic variations. The predominant instances of hemophilia A are linked to the inversion of intron 22 within the FVIII gene. **(C)** Hemophilia B‒associated genetic variations. Missense mutations within the FIX gene account for 68% of hemophilia instances.

Bleeding disorders, such as hemophilia A (HA), hemophilia B (HB), von Willebrand disease (VWD), and a multitude of other bleeding diseases, display a global incidence distribution of 45.8%, 9.9%, 24.7%, and 15.1% for these disorders, respectively (Figure 1A). Most of these disorders are hereditary genetic abnormalities characterized by a reduced physiological ability to produce blood clots, which is crucial for hemostasis, leading to a prolonged period of bleeding after an injury and a heightened risk of hemorrhage in joints or intracranial areas. A comprehensive survey conducted by the World Federation of Hemophilia (WFH) revealed that the annual incidence of bleeding disorders has demonstrated a remarkable increase of 204% from 1999 to 2018 (Stonebraker et al., 2020). In 2023, the global prevalence of hemophilia was estimated at 1,125,000 individuals, including 418,000 males with severe hemophilia (Quintana Paris, 2023). Furthermore, the annual economic burden of severe hemophilia in five European countries in 2014 was approximated at EUR 1.4 billion, equating to around EUR 200,000 per patient (O'Hara et al., 2017).

The diverse manifestations of bleeding disorders present numerous challenges for individuals affected by them. The severity of bleeding is stratified based on the concentrations of coagulation factors, with mild cases showing factor levels from 5 to 40 IU/dL, resulting in prolonged bleeding following surgical operations or injuries, albeit spontaneous bleeding is rarely reported; moderate cases, characterized by factor levels of 1–5 IU/dL, experience occasional spontaneous bleeding and excess bleeding after minor trauma, surgery, and dental extraction; severe cases, identified by a coagulation factor level below 1 IU/dL, endure spontaneous bleeding into joints, muscles, and soft tissues without trauma, along with prolonged bleeding after minor injuries or surgery (Bolton-Maggs and Pasi, 2003; Peyvandi et al., 2016; Srivastava et al., 2020; Castaman et al., 2023). Furthermore, individuals afflicted with severe hemophilia exhibit a heightened susceptibility to the emergence of various complications, such as chronic synovitis, chronic arthropathy, joint deformities, muscular atrophy, and soft tissue contractures (Philipp, 2010; Rodriguez-Merchan, 2010; Zimmerman and Valentino, 2013; Kleiboer et al., 2022).

Hemophilic arthropathy generally manifests as a result of recurrent hemorrhagic episodes within the synovial joints. When hemophilia patients undergo hemorrhaging within a synovial joint, this specific event may induce significant irritation and subsequent damage to the synovium, a specialized membrane that lines the inner surface of synovial joint capsules. Subsequently, the synovial membrane engages in inflammatory responses, leading to pain, swelling, and stiffness in the affected joints. This chronic joint impairment may result in limited physical mobility and a decreased quality of life for individuals afflicted with hemophilia (Gualtierotti et al., 2022; Hauw et al., 2022). The joints of the knees, ankles, and elbows are most affected by hemophilia. Continuous bleeding into these areas can cause joint degeneration, which may eventually affect bone mass and the structures of nearby bones, leading to a high risk of osteoporosis and fractures in hemophilic patients (Rodriguez-Merchan, 2022; Wu and Shen, 2023). The effective management of hemophilia through an integrated medical approach, including factor replacement therapy, joint protection strategies, and the adequate provision of nutritional needs, has the capacity to minimize the detrimental impacts on bone health (Rodriguez-Merchan, 2022; Yen et al., 2022; Lin et al., 2023).

## 2 Common bleeding disorders

Bleeding disorders, specifically HA, HB, hemophilia C (HC), and VWD, are delineated in the following sections.

### 2.1 HA

HA is distinctly characterized by a significant loss of functionality pertaining to the X-linked gene responsible for encoding coagulation factor VIII (FVIII), which plays a critical role in the complex process of blood coagulation essential for hemostasis. The loss of function of the FVIII gene can be attributed to various genetic lesions, with intron 22 inversions being the most common genetic variation, representing nearly 50% of the HA population (Iqbal et al., 2013; Kumar et al., 2015), followed by 16% of small deletions and insertions, 15% of missense mutations, and 10% of nonsense mutations, as well as other causes listed in Figure 1B (Gouw et al., 2012; Franchini and Mannucci, 2022). HA is the most common type of hemophilia, accounting for approximately 80% of all hemophilia cases diagnosed.

### 2.2 HB

HB, commonly referred to as Christmas disease, is distinguished by the loss of function in the X-linked FIX gene. As shown in Figure 1C, missense mutations (68%) and nonsense mutations (14%) within the FIX gene collectively constitute more than 80% of the genetic lesion observed in the population of HB (Shen et al., 2022; Xu et al., 2023; Green et al., 1999). HB makes up an estimated 16% of the overall hemophilia demographic, signifying the second most common variant of hemophilia.

### 2.3 HC

Hemophilia C (HC) constitutes a mere fraction, specifically less than 5%, of the overall hemophilia demographic. This distinct form of hemophilia, also known as plasma thromboplastin antecedent deficiency or Rosenthal syndrome, is marked by the impairment associated with the FXI gene present on chromosome 4. In contrast to HA and HB, which predominantly affect males, HC can affect both genders fairly; however, it is typically considered to demonstrate a reduced severity in terms of hemorrhagic manifestations and their corresponding complications (Cawthern et al., 1998; Jayakrishnan et al., 2019; Kianian et al., 2023).

### 2.4 VWD

VWD is recognized as a hereditary hemostatic disorder stemming from the aberration of the gene residing on chromosome 12 that is essential for the synthesis of von Willebrand factor (VWF), a large multimeric glycoprotein constantly produced by endothelial cells and megakaryocytes (Ruggeri and Ware, 1993; Ruggeri, 2003). The primary role of VWF is to bind with a variety of proteins, particularly FVIII, and it significantly contributes to the process of platelet adhesion at sites of vascular injury and FVIII stability in circulation (Kiouptsi and Reinhardt, 2020; Terraube et al., 2010). Accordingly, FVIII exhibits a half-life of 1–2 h in the absence of VWF, whereas in the presence of fully functional VWF, it is extended to a range of 8–12 h (Terraube et al., 2010).

## 3 Coagulation cascades

**FIGURE 2:**
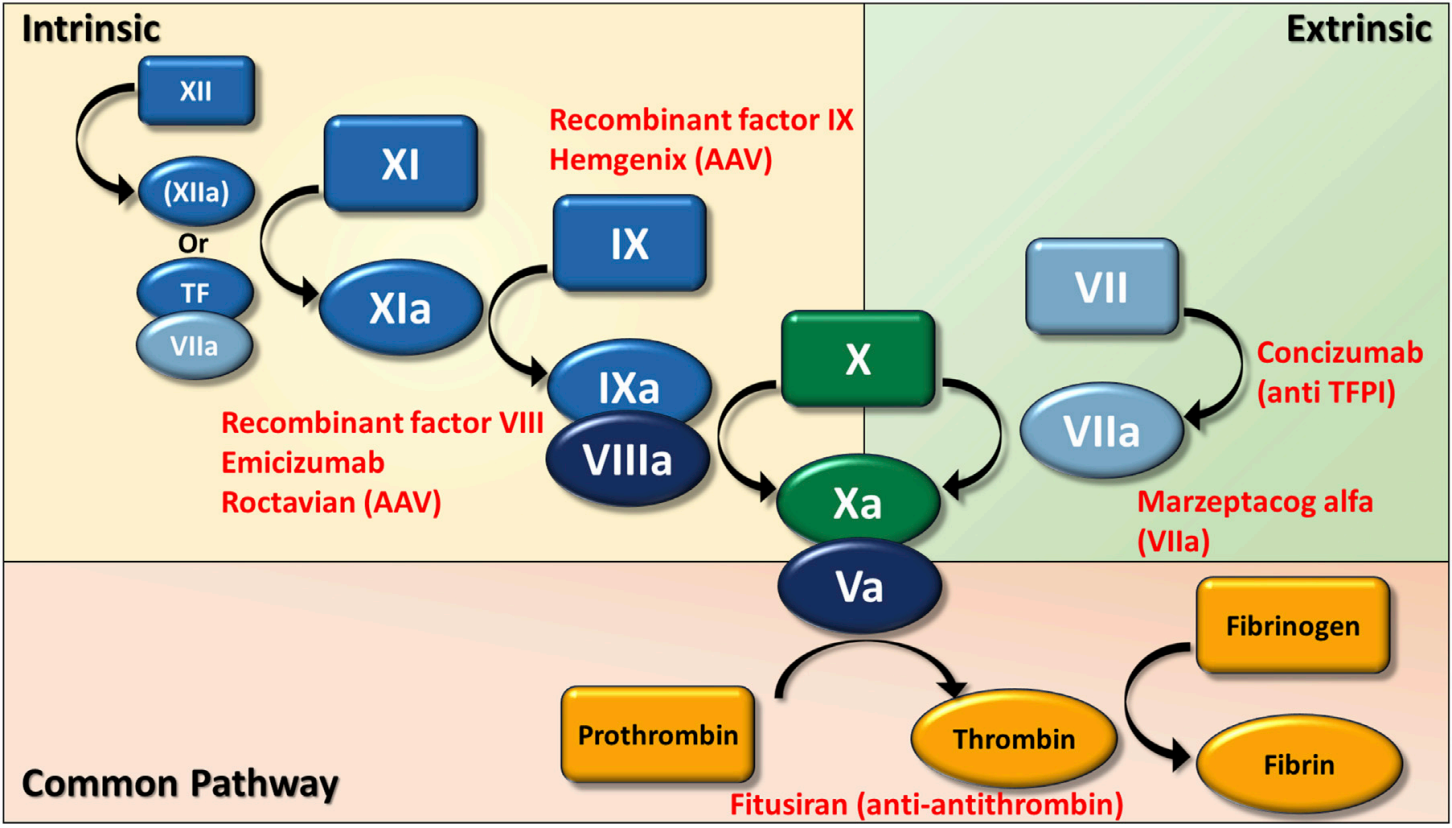
The schematic diagram of the coagulation cascades in conjunction with pharmacological agents targeting specific coagulation factors. The current illustration delineates the connection between the extrinsic coagulation cascade via factor VII and the intrinsic coagulation cascade through factor XII, leading to the common pathway that encompasses the transformation of prothrombin into thrombin, and the ensuing conversion of fibrinogen into fibrin. Pharmacological interventions, including AAV-based gene therapies for the synthesis of recombinant factor IX (Hemgenix) and factor VIII (Roctavian), bispecific antibody directing factor IXa and factor X (Emicizumab), monoclonal antibody against the tissue factor pathway inhibitor (TFPI) (Concizumab), an activated recombinant variant of factor VII (Marzeptacog alfa), and antithrombin-directed siRNA (Fitusiran), have received approval from the United States Food and Drug Administration (FDA) for the prophylaxis or treatment of hemophilia.

As illustrated in Figure 2, the coagulation cascades include extrinsic, intrinsic, and common pathways, which work in concert to form a stable thrombus.

### 3.1 Extrinsic pathway

The extrinsic pathway serves as the initial response by injury to the endothelial tissue (Nakagaki et al., 1991; Mackman et al., 2007). When an injury occurs, tissue factor (TF) is released and binds with activated FVII (FVIIa) to form the TF-VIIa complex, which in turn activates FX to initiate the common pathway that ultimately leads to fibrin formation. The extrinsic pathway is regulated to prevent excessive clot formation through the interaction of FXa with tissue factor pathway inhibitor (TFPI), and the Xa-THPI complex can subsequently inhibit the TF-VIIa complex (Rapaport, 1991; Baugh et al., 1998). This feedback mechanism guarantees the coagulation cascade is meticulously controlled.

### 3.2 Intrinsic pathway

The intrinsic pathway may be initiated through the activation of FXII by means of surface contact or by the TF-VIIa complex via the extrinsic pathway (Wheeler and Gailani, 2016; Satpathy et al., 2025). This process will additionally facilitate the activation of FXI, which subsequently activates FIX. Thereafter, FIXa establishes a complex with FVIIIa to activate FX, which later engages with FVa to connect to the common pathway (Wheeler and Gailani, 2016).

### 3.3 Common pathway

The common pathway integrates both the extrinsic and intrinsic pathways through a complex formed by FXa and FVa. The formed complex functions to facilitate the transformation of prothrombin into thrombin, and thrombin subsequently catalyzes the conversion of fibrinogen into fibrin, thereby establishing a stable hemostatic clot (Kattula et al., 2017).

Overall, the coagulation cascades involve intricate interactions between various factors and pathways. The elaborate orchestration of the coagulation cascades assures a suitable and managed response to tissue trauma, enhancing efficient hemostasis while also mitigating the risk of excessive thrombus formation and hemorrhagic complications. Clinically, the international normalized ratio (INR) calculated using the prothrombin time (PT-INR) is a standardized measurement used to monitor blood coagulation, particularly for patients taking anticoagulant therapy such as warfarin (Walborn et al., 2018). An elevated PT–INR (generally INR ≥1.2) often serves as a criterion for the selection of participants in clinical trials related to sepsis-associated coagulopathy (Vincent et al., 2013). Recently, PT and INR were adopted as indicators for predicting FVII activity in pediatric patients (Bronstein et al., 2023). Although PT and INR demonstrated a degree of correlation with FVII activity, this study was unable to ascertain a definitive cutoff threshold.

## 4 Strategies for hemophilia management

This section comprehensively discusses strategies used for hemophilia management based on clinical trials and preclinical cell and animal studies, dividing the content into protein-based therapies, gene therapy, cell-based therapy, and *in utero* therapy (IUT). Hemophilia provides a relatively forgiving threshold; even modest expression (a few percent of normal factor levels) can have meaningful phenotypic benefit, so moderate delivery efficiency can be therapeutically relevant. Many approved therapeutics, along with some potential investigational drugs undergoing clinical trials, demonstrating favorable clinical results and empirical effectiveness in the management of hemophilia, are elaborated upon in the subsequent discussion, as outlined in Table 1.

**TABLE 1:** Approved and potential investigational therapeutics for hemophilia management.

Category/[citations]	Generic name	Brand name	Manufacturer	Indication^a^	Clinical stage	Approval^b^
Plasma-derived concentrates (PDCs)						
PDC-FVIII + VWF (Kouides et al., 2017; Boban et al., 2024; Federici et al., 2024)	Antihemophilic factor/VWF complex (human)	Humate-P Haemate P	CSL Behring	Prophylaxis and treatment for HA (adult) and VWD (adult and pediatric)		FDA, EMA
	Antihemophilic factor/VWF complex (human)	Wilate	Octapharma	Prophylaxis and treatment for HA (age ≥12 years) and mild/moderate VWD (age ≥6 years)		FDA, EMA
	Antihemophilic factor/VWF complex (human)	Alphanate	Grifols	Prophylaxis and treatment for HA and VWD (adult and pediatric)		FDA
PDC-FVIII (Elalfy et al., 2022)	Antihemophilic factor (human)	Koate-DVI	Kedrion	Prophylaxis and treatment for HA (adult and pediatric)		FDA
PDC-FIX (Hoots et al., 2003; Quon and Logan, 2011; Serban et al., 2012)	Coagulation factor IX (human)	Mononine	CSL Behring	Prophylaxis and treatment for HB (adult and pediatric)		FDA, EMA
	Coagulation factor IX (human)	AlphaNine	Grifols	Prophylaxis and treatment for HB (adult and pediatric)		FDA
	Coagulation factor IX (human)	Haemonine	DHZ Pharma	Prophylaxis and treatment for HB (age ≥6 years)		EMA
Recombinant coagulation factors						
rFVIII (Shapiro, 2007; Turecek et al., 2016; Chowdary et al., 2016; St. Ledger et al., 2018)	Octocog alfa; Antihemophilic factor (recombinant)	Advate	Takeda	Prophylaxis and treatment for HA (adult and pediatric)		FDA, EMA
	Antihemophilic factor (recombinant), PEGylated	Adynovate	Takeda	Prophylaxis and treatment for HA (adult and pediatric)		FDA, EMA
	Antihemophilic factor (recombinant), Fc fusion protein	Eloctate	Biogen Idec	Prophylaxis and treatment for HA (adult and pediatric)		FDA, EMA
	Lonoctocog alfa; Antihemophilic factor (recombinant), single chain	Afstyla	CSL Behring	Prophylaxis and treatment for HA (adult and pediatric)		FDA, EMA
rFIX (Lambert et al., 2007; Ezban et al., 2019; Lyseng-Williamson, 2017)	Nonacog alfa; Coagulation factor IX (recombinant)	BeneFIX	Pfizer	Prophylaxis and treatment for HB (adult and pediatric)		FDA, EMA
	Coagulation factor IX (recombinant), glycoPEGylated	Rebinyn	Novo Nordisk	Prophylaxis and treatment for HB (adult and pediatric)		FDA, EMA
	Albutrepenonacog alfa: Coagulation factor IX (recombinant), albumin fusion protein	Idelvion	CSL Behring	Prophylaxis and treatment for HB (adult and pediatric)		FDA, EMA
Bypassing agents						
aPCC (Ettingshausen et al., 2023)	Anti-inhibitor coagulant complex	FEIBA	Takeda	Prophylaxis and treatment for HA & HB with inhibitor (adult and pediatric)		FDA, EMA
rFVIIa (Shima, 2024)	Coagulation factor VIIa (recombinant)	NovoSeven	Novo Nordisk	Prophylaxis and treatment for HA & HB with inhibitor (adult and pediatric)		FDA, EMA
Non-factor therapeutics						
Bispecific antibody (Shapiro et al., 2018)	Emicizumab	Hemlibra	Roche/Genentech	Prophylaxis rather than acute treatment for HA (adult and pediatric) via subcutaneous route		FDA, EMA
Anti-TFPI antibody (Lamb, 2025; Keam, 2023; Mancuso et al., 2022)	Concizumab	Alhemo	Novo Nordisk	Prophylaxis for HA & HB (age ≥12 years) via subcutaneous route		FDA, EMA
	Marstacimab	Hympavzi	Pfizer	Prophylaxis for HA & HB (age ≥12 years) via subcutaneous route, specifically without inhibitors		FDA, EMA
	Befovacimab (BAY 1093884)		Bayer	Treatment for severe HA & HB via subcutaneous route	Phase 2	
	KN057		Suzhou Alphamab	Prophylaxis for moderate to severe HA & HB via subcutaneous route	Phase 3	
	MG1113		GC Biopharma	Severe HA & HB via subcutaneous route	Phase 1/1b	
siRNA [66–68]	Fitusiran; GalNAc-conjugated siRNA targeting AT mRNA	Qfitlia	Sanofi	Prophylaxis for HA & HB (aged ≥12 years) via subcutaneous route	Phase 3 (For age ≤12 years)	FDA
Viral vector-based Gene therapy						
AAV5-FIX vector (Heo, 2023; Sekayan et al., 2023; Anguela and High, 2024; von Drygalski et al., 2025)	Etranacogene dezaparvovec	Hemgenix	uniQure	One-time treatment for moderate/severe HB (age ≥18 years)		FDA, EMA
AAV5-FVIII vector (Blair, 2022; Ozelo et al., 2022; Mahlangu et al., 2023; Samelson-Jones et al., 2024; Symington et al., 2024)	Valoctocogene roxaparvovec	Roctavian	BioMarin	One-time treatment for severe HA without inhibitors to AAV5 and FVIII (age ≥18 years)		FDA, EMA
AAVRh74-FIX vector (Cuker et al., 2024)	Fidanacogene elaparvovec	Beqvez (Production end in 2025)	Pfizer	One-time treatment for moderate/severe HB without inhibitors to AAVRh74 and FIX (age ≥18 years)		FDA
Autologous stem cells						
Autologous HSCs (Doering et al., 2018; Srivastava et al., 2025)	CD68-ET3-LV CD34^+^			Severe HA	Early phase 1	

^a^ Unless otherwise specified, all drugs were administered intravenously.

^b^ The approval shows only by FDA, and European Medicines Agency (EMA).

### 4.1 Protein-based therapies

#### 4.1.1 Plasma-derived concentrates (PDCs)

PDCs have been used for decades and remain a cornerstone of hemophilia care recognized by the WFH, especially in countries where recombinant options are limited. PDCs are produced through the procedures of plasma pooling from healthy, voluntary donors, which is subsequently advanced by fractionation, purification, and concentration practices to obtain a high yield of the desired factors, in conjunction with viral inactivation protocols to ensure the final products are exceptionally safe. Modern PDCs can be categorized into coagulation factor concentrates, immunoglobulin preparations, and albumin solutions, as well as antithrombin and other protein concentrates, wherein the coagulation factor concentrates, such as FVIII and FIX, are specifically used for the management of HA and HB, respectively.

Earlier, Kouides et al. (2017) analyzed 33 years (1982–2015) of pharmacovigilance data for Humate-P®, a plasma-derived FVIII and VWF concentrate, indicating approximately one adverse drug reaction (ADR) per 3900 administered standard doses. The reported ADRs include low rates of thromboembolic events, inhibitor formation, and hypersensitivity responses, with no confirmed cases of viral or prion transmission, supporting the safety of Humate-P. Mononine® is a plasma-derived FIX concentrate used to treat hemophilia B. The study of Hoots et al. (2003) concluded that continuous intravenous infusion therapy using Mononine is a safe and effective treatment for hemophilia B patients undergoing surgery, experiencing trauma, or severe spontaneous hemorrhage. It effectively maintains therapeutic FIX activity levels and can potentially reduce the overall FIX dosage required, especially when guided by pharmacokinetic assessments.

Other PDCs of FVIII (Koate-DVI®), FIX (AlphaNine®, Haemonine®), as well as FVIII combined with VWF (Wilate®, Alphanate®), have also undergone vigourous evaluation regarding their efficacy and safety in human participants [44–48]. Collectively, most PDCs demonstrate the advantages of being effective, safe, and economically affordable in many regions; however, the exclusive dependence on human donors, the residual risk of infection, variability between batches, inhibitor formation, limited half-life, financial implications and storage constraints, in addition to ethical considerations and supply chain complexities, present significant obstacles to the extensive implementation of PDCs.

#### 4.1.2 Recombinant preparations

Unlike PDCs relying on pooled human plasma donations, recombinant coagulation factors are produced by genetic engineering in mammalian or human cell lines. The purity for recombinant preparations is usually higher than PDCs pertaining to specific factor contents (may be full-length or modified). Infectious risk is virtually nonexistent in patients receiving recombinant preparations because of sterile production conditions. Inhibitor formation is more prevalent with recombinant FVIII (rFVIII) treatment compared to PDCs, though modern products have minimized this (Santagostino et al., 2018). Consistent batch quality is attained through controlled manufacturing processes. Half-life can be extended via PEGylation or Fc fusion to reduce injection frequency and improve patient adherence to prophylactic regimens. The expense for recombinant coagulation factor therapy is typically greater than that for PDCs, though it can differ by region and availability. Recombinant coagulation factors are ideal for prophylaxis and children in developed countries as first-line therapy for hemophilia, in contrast to resource-limited regions using PDCs as the primary treatment strategy.

A variety of recombinant coagulation factors, such as Advate® (rFVIII), BeneFIX® (rFIX), Adynovate® (PEGylated rFVIII), Rebinyn® (PEGylated rFIX), Eloctate® (Fc-fusion rFVIII), Idelvion® (Fc-fusion rFIX), and Afstyla® (single-chain rFVIII), have received FDA approval for HA, HB, or both, as detailed in Table 1 (Shapiro, 2007; Lambert et al., 2007; Turecek et al., 2016; Ezban et al., 2019; Chowdary et al., 2016; Lyseng-Williamson, 2017; St. Ledger et al., 2018).

#### 4.1.3 Bypassing therapy

Bypassing therapy involves the administration of agents that “bypass” the need for the missing coagulation factors, particularly in hemophilic patients who develop neutralizing antibodies (inhibitors) against rFVIII or rFIX replacement products. Bypassing agents are typically administered during acute bleeding events in individuals exhibiting inhibitors, either intraoperatively or postoperatively, and sometimes as a prophylactic measure aimed at decreasing the incidence of bleeding in patients with inhibitors (Leissinger et al., 2015). 20%–30% of those with HA and 3%–10% of HB develop inhibitors to factor VIII and IX, making standard treatments ineffective (Butros et al., 2011).

Two types of classic bypassing agents are used in clinical practices, namely, activated prothrombin complex concentrate (aPCC) and recombinant activated factor VII (rFVIIa). FEIBA® is a bypassing agent of the aPCC type, which contains multiple coagulation factors (mainly FVII, FIX, FX, and FVIIa) and has the capability to initiate the clotting cascade in the absence of FVIII or FIX. The recent FEIBA GO study has demonstrated the sustained, real-world efficacy and a reliable safety profile of aPCC when administered as both an on-demand therapeutic intervention and a prophylactic measure in patients with hemophilia and high-titer inhibitors (Ettingshausen et al., 2023). The representative rFVIIa, NovoSeven®, functions by directly activating FX to FXa on activated platelet surfaces, by passing FVIII and FIX, thus facilitating thrombin generation and clot formation in patients with inhibitors. NovoSeven has been extensively utilized worldwide for the management of hemorrhagic episodes in patients with diverse rare coagulopathies such as congenital HA or HB with inhibitors, acquired hemophilia, congenital factor VII deficiency, and Glanzmann thrombasthenia with demonstrated efficacy (Shima, 2024). Both aPCC and rFVIIa are associated with an elevated risk of thrombosis and disseminated intravascular coagulation, thereby necessitating meticulous monitoring for signs of thromboembolic events during their administration (Ettingshausen et al., 2023; Shima, 2024).

#### 4.1.4 Alternative therapeutics

The emergence of non-factor-based therapies has presented alternative treatment approaches and mechanisms of action that are distinct from existing bypassing agents. Emicizumab (Hemlibra®, Food and Administration (FDA) approved in 2017), a bispecific monoclonal antibody that mimics the scaffolding function of FVIIIa by bridging FIXa and FXa, is the first non-factor-based prophylaxis agent for inhibitor patients; however, its use necessitates that clinicians reassess their methodologies for handling breakthrough bleeds and tackle the primary cause of inhibitors (Shapiro et al., 2018). Anti-TFPI monoclonal antibodies, categorized as another non-factor-based prophylactics, function by obstructing TFPI’s inhibitory effects on FXa, thereby restoring equilibrium in the coagulation cascade. Two emerged products of anti-TFPI monoclonal antibodies are currently approved to prevent or reduce bleeding episodes, marstacimab (Hympavzi®, approved in the US and Europe) (Lamb, 2025) and concizumab (Alhemo®, approved in Canada) (Keam, 2023), while their long-term data are still accumulating. Other similar agents, like Befovacimab (Mancuso et al., 2022), KN057 (Phase 3, ClinicalTrials.gov identifier: NCT06569108), and MG1113 (Phase 1: NCT03855696), are currently in extensive clinical evaluation.

Antithrombin (AT, encoded by *SERPINC1*) is an endogenous anticoagulant that prevents excessive clotting by neutralizing several activated coagulation factors, mainly thrombin and FXa, and to a lesser extent FIXa, FXIa, and FXIIa, maintaining balance between coagulation and anticoagulation (Rodgers and Mahajerin, 2023). Application of AT-directed small interfering RNA (siRNA) agents, such as fitusiran (Qiftlia®) (Machin and Ragni, 2018), represents another prophylactic approach to manage bleeding episodes in hemophilia by inhibiting the translation of AT mRNA in the liver, leading to the reduction in plasma AT level. Fitusiran is the first FDA-approved siRNA therapy (administered subcutaneously once monthly) by demonstrating positive effects from randomized controlled Phase III clinical trials across different types of hemophilia regardless of whether a patient has FVIII or FIX deficiency or the presence of plasma inhibitors (Lu et al., 2025). Despite the successful application of fitusiran, it is vital to address the various adverse effects, which include considerable thrombotic incidents resulting from excessive reduction of AT levels, minor to moderate rises in hepatic ALT and AST concentrations, injection site reactions, and general systemic effects (Pipe et al., 2025). Therefore, it is essential to meticulously oversee AT levels, regularly assess liver function, and adjust treatment in conjunction with factor concentrates or bypassing agents for the effective utilization of fitusiran.

### 4.2 Gene therapy

#### 4.2.1 Viral gene therapy

Gene therapy for hemophilia aims to provide a long-lasting or potentially curative treatment by enabling the patient’s own cells to produce the missing clotting factor (FVIII or FIX), instead of relying on regular infusions of replacement factor proteins. As of now, two available adeno-associated virus serotype 5 vector (AAV5)-based gene therapies have been authorized for hemophilia (Pierce and Herzog, 2023). Etranacogene dezaparvovec (Hemgenix®), which incorporates the *Padua* variants of FIX, has received approval in the US, Canada, and Europe for use in adults with moderate to severe hemophilia B (Heo, 2023; Sekayan et al., 2023; Anguela and High, 2024; von Drygalski et al., 2025). Valoctocogen roxaparvovec (Roctavian®), another gene therapy delivering the FVIII gene, has received European approval for adults with severe HA, contingent upon the absence of detectable FVIII inhibitors or specific pre-existing antibodies to the AAV5 vector (Blair, 2022; Ozelo et al., 2022; Mahlangu et al., 2023; Samelson-Jones et al., 2024; Symington et al., 2024). Fidanacogene elaparvovec (Beqvez®), akin to Hemgenix, received US approval in 2024 for hemophilia B (Cuker et al., 2024), but was later retracted by its manufacturer in early 2025 for commercial reasons. Both Hemgenix and Roctavian therapies show significant increases in circulating FVIII or FIX levels (often 10–50% of the normal range) and reductions in annualized bleeding rate (ABR) in multiyear follow-up studies, as well as common adverse effects, such as elevation in liver enzymes (ALT, AST), and other frequent side effects (nausea, headache, infusion-related reactions, etc.) have been reported (von Drygalski et al., 2025; Symington et al., 2024). In the context of durability, Hemgenix therapy led to the FIX activity levels remaining relatively stable from year 1 through year 4 with slight fluctuations but no major drop-off (von Drygalski et al., 2025); however, Roctavian therapy caused FVIII levels to tend to decline from early highs to lower levels by years three to five in many patients, with the median durability ranging from 7.2 years to 31.8 years (Symington et al., 2024).

#### 4.2.2 Non-viral gene therapy

To date, several non-viral strategies have been evaluated in animal models of hemophilia. These strategies include lipid nanoparticle (LNP)-mediated delivery of CRISPR/Cas9 systems (Han et al., 2022; Han et al., 2023; Lee and Han, 2024; Chen et al., 2024), transposon systems (Liu et al., 2006; Kren et al., 2009; Staber et al., 2014; Di Matteo et al., 2014; Mats et al., 2014), hydrodynamic gene delivery, and various chemical/polymeric carriers and targeted nanoparticles. These systems need to protect nucleic acids from degradation, promote cellular uptake and endosomal escape, and ideally target the liver specifically to reduce off-target transfection and toxicity. The above non-viral approaches can be used alone or together in practice, but most are still in the preclinical stage for hemophilia.

LNPs carrying Cas9 mRNA and small guide RNA (sgRNA) or related editors have been used to edit the *SERPINC1* gene in the liver to rebalance coagulation and correct the bleeding phenotype in HA/HB mice (Han et al., 2022). The studies produced a durable reduction of AT and improved thrombin generation and bleeding outcomes after single systemic doses. This is a benchmark proof-of-concept that non-viral LNP editing can treat hemophilia by pathway rebalancing rather than direct factor replacement. Follow-up studies by the same group optimized LNP chemistry and dosing (combination of low-dose AAV and LNP approaches) to improve efficacy while lowering viral load when needed, showing sustained therapeutic thresholds in their mouse models (Han et al., 2023). Furthermore, the LNP-mediated CRISPR/Cas9 system has been shown to successfully induce the correction of the mutant FVIII gene in HA mice with sustained FVIII activity up to 6% over 26 weeks and an average gene editing rate of 15.3% in liver sinusoidal endothelial cells (LSECs), offering a promising alternative to current treatments (Chen et al., 2024).

Transposon systems, such as Sleeping Beauty (SB) and piggyBac (PB), have been engineered to facilitate the delivery of non-viral plasmids or nanoparticles containing transposons and transposases for the stable integration and sustained expression of target genes. SB transposon-based strategies have been shown to produce therapeutic FVIII expression up to 50 weeks in HA mice (Liu et al., 2006; Kren et al., 2009). PB-based systems can carry large cDNAs (important for full-length FVIII) and support long-term expression after genomic insertion—attractive for HA and HB. Studies reported therapeutic or near-therapeutic FIX/FVIII levels and reduced bleeding in mice, alongside sustained expression for months and hyperactive PB transposases enhancing integration efficiency and persistence (Staber et al., 2014; Di Matteo et al., 2014; Mats et al., 2014). However, there appears to be a gap in follow-up research reports regarding the use of transposon systems for treating hemophilia over the last 10 years.

Hydrodynamic tail-vein (HTV) injection delivers naked plasmid/minicircle DNA efficiently to mouse hepatocytes by a rapid, large-volume intravenous bolus (commonly ∼8–10% of mouse body weight in seconds). It is widely used to evaluate hepatic expression of FIX or FVIII and to test genome-editing strategies *in vivo* (Ye et al., 2003; Keravala et al., 2011; Chavez et al., 2014; Kamimura et al., 2023). Of these studies, HTV injection of plasmids encoding ΦC31 integrase and hFIX/hFVIII led to genomic integration at pseudo-*attP* sites, durable FIX/FVIII expression for over 6 months, and achieved phenotypic correction (Keravala et al., 2011; Chavez et al., 2014). This shows HTV can be combined with site-specific integrases for durable nonviral expression. HTV delivery serves as a promising proof-of-concept in rodent models, but significant modifications and adaptations in larger animals are essential for its clinical applicability in humans. Recently, a preclinical trial in baboons demonstrated the safety and effectiveness of liver-specific hydrodynamic gene delivery for hemophilia gene therapy (Kamimura et al., 2023). The results from this study validated targeted gene delivery and sustained hFIX expression in baboon liver for 100–150 days, achieving coagulation activity above the therapeutic threshold of 10%, while also demonstrating consistent results after repeat administrations 210 days post-initial injection with hFIX expression levels 5–10 times greater than the initial dose. Furthermore, minicircles often outperform standard plasmids for sustained expression because they lack bacterial CpG motifs and the plasmid backbone that promotes silencing, thereby facilitating preclinical assessments of minicircles for enhanced FVIII/FIX expression with lower inflammatory responses (Chanani et al., 2025).

A multitude of chemical/polymeric carriers and targeted nanoparticles have been tested for hepatic delivery of plasmids, mRNA, and ribonucleoproteins encoding FIX and FVIII, and their efficacy and toxicity depend heavily on formulation. These cases include lipid-like nanoparticles (lipidoids) (DeRosa et al., 2016), chitosan (cationic polysaccharides) nanoparticles (Bowman et al., 2008; Quade-Lyssy et al., 2014), biodegradable polycations (polyethylenimine (PEI), poly-β-amino ester (PBAE), and relatives) (Th et al., 2019), and surface ligand or protein corona conjugated nanoparticles (Wahane et al., 2020). Of these, lipidoids carrying hFIX mRNA showed robust transient protein expression after intravenous dosing and have advanced into primate testing (DeRosa et al., 2016); oral delivery of chitosan nanoparticles carrying hFVIII and FIX plasmid/minicircles showed transient systemic expression after repeated dosing and partial phenotypic correction with generally low and variable efficiency (Bowman et al., 2008; Quade-Lyssy et al., 2014); PBAEs showed promising low toxicity and efficient DNA compaction and endosomal escape *in vivo*, and high-throughput polymer libraries have been screened to find liver-tropic formulations (Th et al., 2019); surface ligands, including N-acetylgalactosamine (GalNAc), peptides, and antibodies, and protein corona engineering with ApoE are used to bias nanoparticles to hepatocytes or LSECs (Wahane et al., 2020).

Considering the issues of safety, limitations, and translational gaps, LNPs for liver delivery show promising tolerability but require detailed immunology and off-target editing assessment prior to clinical translation; transposon/integration strategies pose a risk of insertional mutagenesis that needs to be elucidated through extensive studies in large-animal models; transient approaches (mRNA/RNP via LNP) reduce long-term nuclease exposure but may require re-dosing; hydrodynamic injection is largely a rodent technique; LNPs encounter challenges related to scalability, biodistribution, and immune responses in larger animals and humans that must be addressed in primate studies.

### 4.3 Cell-based therapy

#### 4.3.1 LSEC transplantation

Prior studies have established that the transplantation of LSECs can restore the liver endothelium and mitigate the phenotypic expressions in HA mice (Kumaran et al., 2005; Follenzi et al., 2008). LSECs are now recognized as the main physiologic source of FVIII in adults (not hepatocytes) (Shahani et al., 2014), thereby engrafting FVIII-producing LSECs from healthy donors is expected to restore circulating FVIII. Building upon this concept, Gage et al. (Gage et al., 2022) protocoled to make LSEC-like cells from human pluripotent stem cells (PSCs) and transplanted them into immunocompromised HA mice, showing FVIII secretion and phenotypic rescue. Following this, Mitani et al. (Mitani et al., 2023; Mitani et al., 2024) demonstrated scalable production of LSEC progenitors from human bone marrow-derived mesenchymal stem cells (MSCs) and induced PSCs (iPSCs), providing prospective cell-based approaches for hemophilia management. Moreover, Saiki et al. showed iPSC-derived FIX-secreting hepatocyte sheets and liver organoids for modelling/treatment.

#### 4.3.2 Autologous stem cell transplantation

Autologous stem cell transplantation herein for hemophilia associates with *ex vivo* gene correction using CRISPR/Cas9 editing or AAV and lentivirus (LV) vector -based gene transduction in autologous iPSCs, MSCs, and hemopoietic stem cells (HSCs), and, followed by the transplantation of these corrected cells back into the same individual to enable normal expression of FIX and FVIII. This approach holds advantages in the lack of immune rejection and the potential of long-term engraftment with stable FIX or FVIII synthesis.

Several proof-of-concept studies highlight the therapeutic gains in murine models of hemophilia employing CRISPR/Cas9-modified stem cell transplantation. For example, Park et al. (2015) established a robust method for correcting intron 22 inversion underlying severe HA using CRISPR/Cas9 in patient-derived iPSCs. Their results demonstrated functional rescue observed in HA mice (10% of that in wild-type mice), coupled with the absence of detectable off-target mutations and maintenance of pluripotency, position this approach as a promising avenue for future cell-based therapies for hemophilia and other genetic disorders involving large chromosomal rearrangements. Similarly, Lyu et al. (2018) underscored the feasibility of generating patient-specific, FIX-corrected iPSCs and their differentiation into functional hepatocytes for potential therapeutic use for hemophilia B, and Rose et al. (2020) demonstrated the potential of patient-specific iPSC-derived endothelial cells as a viable cell therapy for HA, capable of sustained FVIII delivery and symptom amelioration in animal models, paving the way for future clinical applications. Furthermore, Qiu et al. (2021) demonstrated the transplantation of induced MSCs (iMSCs) derived from FVIII-corrected patient iPSCs into HA mice successfully restored FVIII function and rescued the HA phenotype; however, plasma FVIII activity was sustained for only 3 weeks in HA mice, possibly due to the development of immunogenicity against human cells in immunocompetent HA mice.

MSCs can differentiate into chondrocytes and osteoblasts, and produce various bioactive mediators, suggesting their potential to repair degenerative joints and modulate inflammatory responses, which are key in the pathophysiology of hemophilic arthropathy. The early study of Kashiwakura et al. (2012) revealed that a single intra-articular injection of LV-transduced MSCs expressing hFVIII markedly improves hemarthrosis and hemophilic arthropathy in HA mice for at least 8 weeks; despite the promising results, the procedure induced a low inhibitor titer, which might enhance immune responses to hFVIII in patients with inhibitors, suggesting further evaluations in larger animals and long-term safety assessments are required. Afterward, Ohmori et al. (2018) provided strong evidence for the long-term (16 months) safety of intra-articular transplantation of LV-transduced hFVIII-expressing MSCs in non-human primates, highlighting the absence of tumor formation and systemic proviral integration. This finding is crucial for advancing the procedure towards clinical application. Human umbilical cord MSCs (HUCMSCs) serve as a promising reservoir for cell-based therapy. Bu et al. (2024) recently developed an approach for HB, in which they found that HUCMSCs transduced with a scAAV-hFIX vector demonstrated robust secretion of hFIX over a 5-month period in HB mice, with no clonal expansions of the transduced cells reported in mice, indicating a favorable safety profile.

HSC-derived approaches are definite proof-of-concept in animal models, and now early human trials show some success for HA using autologous LV-transduced HSCs. Doering et al. (2018) developed a genetically modified autologous cell product, designated CD68-ET3-LV CD34^+^, for the treatment of severe HA. A unique aspect of CD68-ET3 LV is the inclusion of the CD68 promoter, which directs monocyte/macrophage-specific expression. The bioengineered FVIII transgene (ET3) demonstrated superior biosynthetic efficiency compared to B domain-deleted (BDD) human FVIII, suggesting higher potency for the CD68-ET3-LV CD34^+^ product. This product demonstrated its effectiveness works by HSC engraftment, differentiation into blood cell lineages, and the subsequent secretion of FVIII into the bloodstream in HA mice. These preclinical findings provide a strong rationale for advancing CD68-ET3-LV CD34^+^ to Phase 1 clinical trial (NCT05265767). The early results from NCT05265767 demonstrated the gene therapy using lentiviral vector-transduced autologous HSCs for severe HA showing stable and therapeutically significant FVIII expression, effectively preventing bleeding with a favorable safety profile (Srivastava et al., 2025). The unique design elements of the vector contribute to its efficacy, and the initial results are promising for broad application, potentially even at an early age, with reduced concerns regarding infertility due to the conditioning regimen. At present, a comparable Phase 1 clinical trial (NCT04418414) has been documented for implementation, but participant recruitment has not yet begun. In addition, with the advancement of the αIIb promoter, alternative techniques associated with platelet-directed LV (2bF8, 2bF9)-mediated gene delivery to HSCs have been developed for their transplantation to murine models of HA and HB (Shi et al., 2007; Chen et al., 2014; Kumar et al., 2024). Given that HSCs possess the capacity to differentiate into platelets, the related studies collectively underscore the efficacy of platelet-targeted expression of FIX and FVIII in ameliorating the bleeding phenotype and fostering antigen-specific immune tolerance in both HA and HB murine models, notwithstanding the presence of pre-existing immunity.

### 4.4 In utero therapy (IUT)

The concept of IUT for hemophilia refers to the administration of therapeutic interventions to the fetus prenatally, employing methodologies such as viral vector-mediated gene therapy, stem cell transplantation, or an integrative approach incorporating both techniques, thereby facilitating the early synthesis of therapeutic FVIII or FIX to avert bleeding and promote immune tolerance. This innovative practice is attractive due to some reasons, including (1) the limited size of the fetus—leading to a markedly lower necessary dosage of vectors or cells relative to postnatal therapy (Porada et al., 2014), (2) the tolerogenic nature of the fetal immune system—enhancing the likelihood of diminishing anti-vector or anti-factor immune responses (Almeida-Porada et al., 2016), and (3) enduring protection—early intervention could prevent bleeding episodes, joint deterioration, and the need for lifelong prophylaxis (Mattar et al., 2023).

Diving into the early animal works is exciting because they show how IUT has been explored from the ground up and become one of the viable treatment options of the future. The initial proof-of-concept study of IUT employing viral vector-mediated gene therapy for hemophilia was performed by Waddington et al. (2003), where an adenoviral vector carrying hFIX was administered intravenously (via the yolk sac-associated vitelline duct) to fetal mice. The findings indicated that certain mice exhibited sustained hFIX expression after birth, notably without developing anti-hFIX antibodies, contrasting with controls that exhibited robust antibody responses. While the study did not fully correct a hemophilia phenotype, it was a landmark demonstration that *in utero* transfer in mice is feasible and has immunologic consequences of tolerance induction. Subsequently, Mattar et al. (2011) demonstrated that a single intravenous injection of AAV vectors (AAV5 and AAV8, 4 × 10^12^ copies/animal) with hFIX in late-gestation non-human primate fetuses could achieve long-term therapeutic protein expression (up to 22 months) without the need for re-administration in early infancy. The expression of hFIX was liver-specific and did not trigger a neutralizing immune response; additionally, AAV8 induced higher hFIX expression and a milder immune response compared to AAV5, and there was no maternal germline transmission. Furthermore, through the utilization of lentiviral vectors, Kao et al. (2016) demonstrated that prenatal administration of the ubiquitous chromatin opening element from the human *HNRPA2B1-CBX3* housekeeping gene locus (A2UCOE)-based LV vectors (2 × 10^7^ copies/animal) to the fetal liver is an effective strategy. Their approach efficiently transduces both hepatocytes and HSCs in the murine fetal liver. Even in the context of a relatively low average vector copy number (0.19 per liver cell), the A2UCOE-FIX vector succeeded in yielding stable levels of plasma FIX production over a long period of 7 months, which were adequate to ameliorate severe HB (<1%) to a mild phenotype (approximately 20% of normal FIX levels), a result considered therapeutic. The method of Kao et al. is considered a viable alternative to high-dose AAV vector delivery for treating HB and other metabolic conditions requiring therapeutic gene delivery to the liver.

**FIGURE 3:**
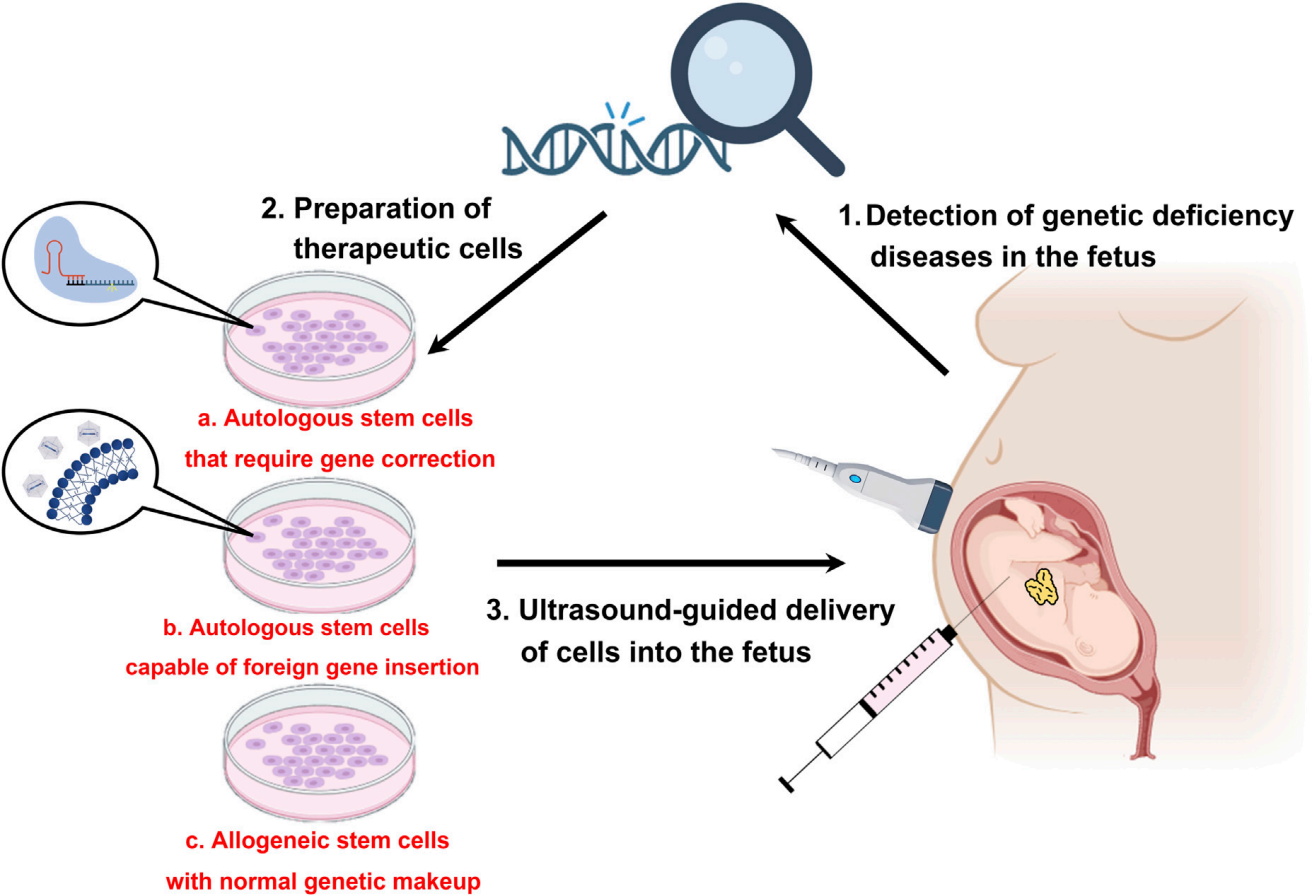
*In utero* stem cell therapy. The central concept of *in utero* stem cell therapy is built upon the recognition of congenital anomalies present in the fetus, succeeded by the *ex vivo* rectification of the genetic abnormalities in either autologous or allogeneic stem cells, culminating in the ultrasound-mediated delivery of the corrected stem cells into the fetus to ensure normal developmental processes. This intervention also possesses the capacity to condition the fetal immune system, thereby augmenting immune tolerance.

IUT may also be conducted via the transplantation of genetically engineered stem cells into the fetus. The main procedures involve the collection of stem cells, *ex vivo* expansion of the collected stem cells, and precise intrauterine transplantation of the expanded stem cells (Figure 3). Therapeutic stem cells may be autologous, necessitating genetic correction through viral (AAV or LV) or non-viral methods (like LNP-CRISPR/Cas9), or allogeneic, which avoid genetic alteration but risk fetal rejection; these cells can be administered to the fetus under ultrasound guidance to aid in its development. The study conducted by Rosen et al. (2003) signifies the pioneering research utilizing *in utero* transplantation aimed at treating hemophilia, wherein they tackled a lethal perinatal bleeding disorder (FX deficiency) by transplanting wild-type fetal liver cells into mid-gestation mouse embryos. Kumar et al. (2018) demonstrated the viability of utilizing genetically engineered (BDD-FVIII expression) placenta-derived mesenchymal stromal cells (PMSCs) for *in utero* transplantation as a prospective treatment for HA, capitalizing on the distinctive benefits of the fetal environment, while indicating the potential to isolate PMSCs from first-trimester chorionic villus tissue via chorionic villus sampling, thus facilitating early HA diagnosis and cell collection for autologous therapy. Kao et al. (2023) developed a novel *in utero* cell therapy for HA mice using human amniotic fluid mesenchymal stromal cell (hAFMSC) engraftment, demonstrating successful integration of transplanted cells within the recipient liver (one human cell per 10,000 mouse cells), sustained FVIII activity throughout a 12-week observation period after birth, and minimal inhibitor levels detected during the study period. Moreover, Rodriguez et al. (2023) illustrated the viability, immunological benefits, and safety of prenatal transplantation of human placental cells transduced (10^7^–10^8^/kg) with a bioengineered FVIII transgene (PLC-mcoET3) in fetal sheep for hemophilia A treatment. Their findings indicated significant and prolonged increases in plasma FVIII levels for over 3 years post-transplant, achieving levels that could lead to normal hemostatic function. The recipients did not produce anti-FVIII inhibitory antibodies or immune responses against the transplanted cells or the FVIII they synthesized. The persistence of PLC-mcoET3 engraftment was verified in multiple tissues, including liver, lung, spleen, and thymus, enduring for years after birth; however, no indications of lentiviral-related toxicity, alterations in tissue architecture, or tumor formation were observed. While these studies present promising results, future research needs to address the risks associated with cell transformation, optimize culturing conditions, ensure long-term monitoring, and potentially improve engraftment efficiency for broader clinical applicability. To our current understanding, there are no approved human prenatal gene or cell therapies available for the treatment of hemophilia at this moment.

Notwithstanding the numerous benefits associated with IUT, considerable safety concerns, technical challenges, and ethical dilemmas remain to be resolved. In the context of safety concerns, procedural risks posed to both the fetus and the mother are of utmost importance. Any needle or fetoscopic intervention (such as amniocentesis, intraperitoneal injection, or fetoscopy) entails a minor yet significant risk of miscarriage, fetal bleeding, or the onset of preterm labor, alongside potential maternal complications (such as bleeding and infection), with these risks being augmented by gestational age, access route, and the practitioner’s expertise (Sagar et al., 2020). Ectopic engraftment may lead to functional impairment, localized inflammation, or abnormal tissue proliferation (Sagar et al., 2020). The risk for neoplastic transformation is existed when modifications to cellular genomes occur through genetic engineering (Mattar et al., 2024). Even non-integrating approaches have theoretical risks if cells acquire mutations or stimulate oncogenic pathways, and long latency makes detection challenging without long-term follow-up. Although the goal is somatic correction, fetal interventions potentially reach germ cells, especially very early gestational interventions, raising heritable alteration concerns and strong regulatory and ethical objections (Lynn et al., 2024). Transplanted cells or immune/inflammatory responses may influence organogenesis, neurological maturation, or growth, necessitating extensive follow-up over decades for many potential late outcomes (Ishii and Eto, 2014). In the realm of technical challenges, transplanted cells might encounter unsuccessful engraftment, be surpassed by native fetal progenitor cells, succumb post-transplantation, or yield inadequate levels of FVIII/FIX—leaving the child untreated while having exposed the mother/fetus to risk; preclinical models frequently demonstrate partial or time-limited correction (Almeida-Porada et al., 2016). Maternal exposure to donor antigens poses a risk of sensitization, potentially leading to hemolytic disease in subsequent pregnancies or complicating transfusion outcomes, while fetal immune development may exhibit either tolerogenic or immunogenic responses influenced by cell type, dosage, and timing, with the possibility that anti-factor antibodies may neutralize postnatal therapeutic benefits (Sagar et al., 2020). The transmission of pathogens via donor cells or production contamination, coupled with maternal infection risks, underscores the imperative for rigorous donor screening and compliance with Good Manufacturing Practices (GMP) (Sagar et al., 2020). A comprehensive longitudinal evaluation of tumorigenesis, reproductive effects, immune responses, and functional longevity is imperative; however, the obligation for ongoing evaluation (clinicians, sponsors, health systems) is still not well-defined, thereby requiring extensive Good Laboratory Practice (GLP)-compliant large animal data prior to the onset of human trial activities (Hendriks et al., 2024). In ethical considerations, the fetus’s inability to provide consent necessitates parental decision-making amidst uncertainties regarding long-term implications, while issues of social justice and equitable access to early interventions further exacerbate ethical dilemmas (Hendriks et al., 2024).

## 5 Future perspectives and challenges

Hemophilia clinical treatments have made major strides, with advancements in cell therapy and gene therapy offering novel approaches to address the challenges associated with this genetic bleeding disorder. The cost of hemophilia treatment has been a longstanding concern. However, cell therapy and gene therapy present opportunities for substantial improvements. While these innovative treatments may initially involve high development costs, the potential for long-term cost reduction becomes apparent when considering the prospect of fewer factor replacement infusions and associated medical interventions. Moreover, the elimination of routine replacement injections can enhance the quality of life resulting from sustained and endogenous clotting factor production.

Exploring cell therapy *in utero* represents a forward-thinking strategy. Giving stem cell-based treatments during fetal development could change how the immune system reacts to clotting factors early on. Even if the immediate therapeutic effects are not fully realized, proactive education of the immune system during fetal stages could contribute to reducing the likelihood of developing neutralizing antibodies against clotting factors later in life. The concept of leveraging early interventions for long-term benefits is in line with this preventive approach.

In forthcoming research, the trajectory of *in utero* stem cell transplantation should prioritize enhancements in targeted delivery mechanisms to the fetal liver or LSECs, while simultaneously mitigating off-target effects and germline exposure; comprehensive large-animal safety evaluations (akin to GLP) and the establishment of standardized long-term monitoring protocols to gauge developmental and reproductive safety; as well as investigations into cell sources (such as fetal LSECs and engineered placental cells) and *ex vivo* modification techniques that facilitate controlled interventions prior to *in utero* application. These efforts will be crucial for optimizing therapeutic strategies and ensuring the safety and efficacy of *in utero* interventions, ultimately improving outcomes for affected fetuses. Future studies must also explore the integration of minimally invasive techniques to enhance the feasibility of these interventions, thereby furthering the potential of *in utero* therapies for congenital disorders. These advancements will not only improve therapeutic efficacy but also address ethical concerns surrounding *in utero* interventions, ensuring a holistic approach to fetal treatment.

## 6 Conclusion

The future of hemophilia clinical treatment appears promising, driven by the potential of cell therapy and gene therapy to revolutionize care. Balancing the equation of cost and quality, coupled with innovative strategies like *in utero* interventions, showcases a holistic perspective that extends beyond the conventional boundaries of treatment. As research progresses and clinical trials unfold, the journey toward more effective, accessible, and sustainable hemophilia treatments continues to evolve.
